# Supplementary material for: The perceptual neural trace of memorable unseen scenes
Source: Sci Rep. 2019 Apr 15;9:6033. doi: 10.1038/s41598-019-42429-x (PMC6465597; doi:10.1038/s41598-019-42429-x)
Supplement: Supplementary file 1 — Supplementary Info [file 41598_2019_42429_MOESM1_ESM.docx]

**The perceptual neural trace of memorable unseen scenes**

Yalda Mohsenzadeh^1,2^, Caitlin Mullin^1^, Aude Oliva^1^, Dimitrios Pantazis^2^

1- Computer Science and Artificial Intelligence Lab., Massachusetts Institute of Technology, Cambridge, MA, USA

2- McGovern Institute for Brain Research, Massachusetts Institute of Technology, Cambridge, MA, USA

**Supplementary Information**

***Stimulus set features control***

As described in the Methods section, the 30 scenes comprised of 15 high memorable and 15 low memorable images controlled for low level features (color, luminance, brightness, and spatial frequency) using the natural image statistical toolbox^1-2^. The toolbox provides scripts to: i) statistically compare spatial frequency (spectral energy) information between two image sets. The results of this comparison between high and low memorable scene images of our stimulus set in four energy levels of 10%, 30%, 70%, and 90% are presented in Table S1. As demonstrated in Table S1, the two sets are not statistically different in terms of spatial frequency. ii) statistically compare color distribution information between two image sets. The results of this comparison between high and low memorable scene images in two color spaces, RGB and Lab, are presented in Table S2. As shown, we controlled the two stimulus conditions for color, brightness, and contrast. These low-level features are useful for controlling activation in early visual cortex.

Furthermore, we controlled for high level categorical semantics between the two sets. Each set includes scenes containing food, animals, vehicles, flowers, and objects. The stimulus set is available at <https://memorabilityrsvp.github.io>.

**Table S1.** Comparison of spatial frequency information between high and low memorable image sets.

|  | **Spectral Energy Level (Spatial Frequency)** | | | |
| --- | --- | --- | --- | --- |
|  | **10%** | **30%** | **70%** | **90%** |
| **t value** | -0.43 | -0.99 | -0.88 | -1.03 |
| **p value** | 0.66 | 0.32 | 0.38 | 0.31 |

**Table S2.** Comparison of color distribution information between high and low memorable image sets.

|  | **Color Distribution** | | | | | |
| --- | --- | --- | --- | --- | --- | --- |
|  | **RGB Space** | | | **Lab Space (Brightness and Contrast)** | | |
|  | R | G | B | L | a | b |
| **t value** | -0.53 | 0.04 | 0.95 | -0.23 | -0.02 | -1.49 |
| **p value** | 0.59 | 0.96 | 0.34 | 0.81 | 0.97 | 0.14 |

***MEG dSPM Cortical Maps***


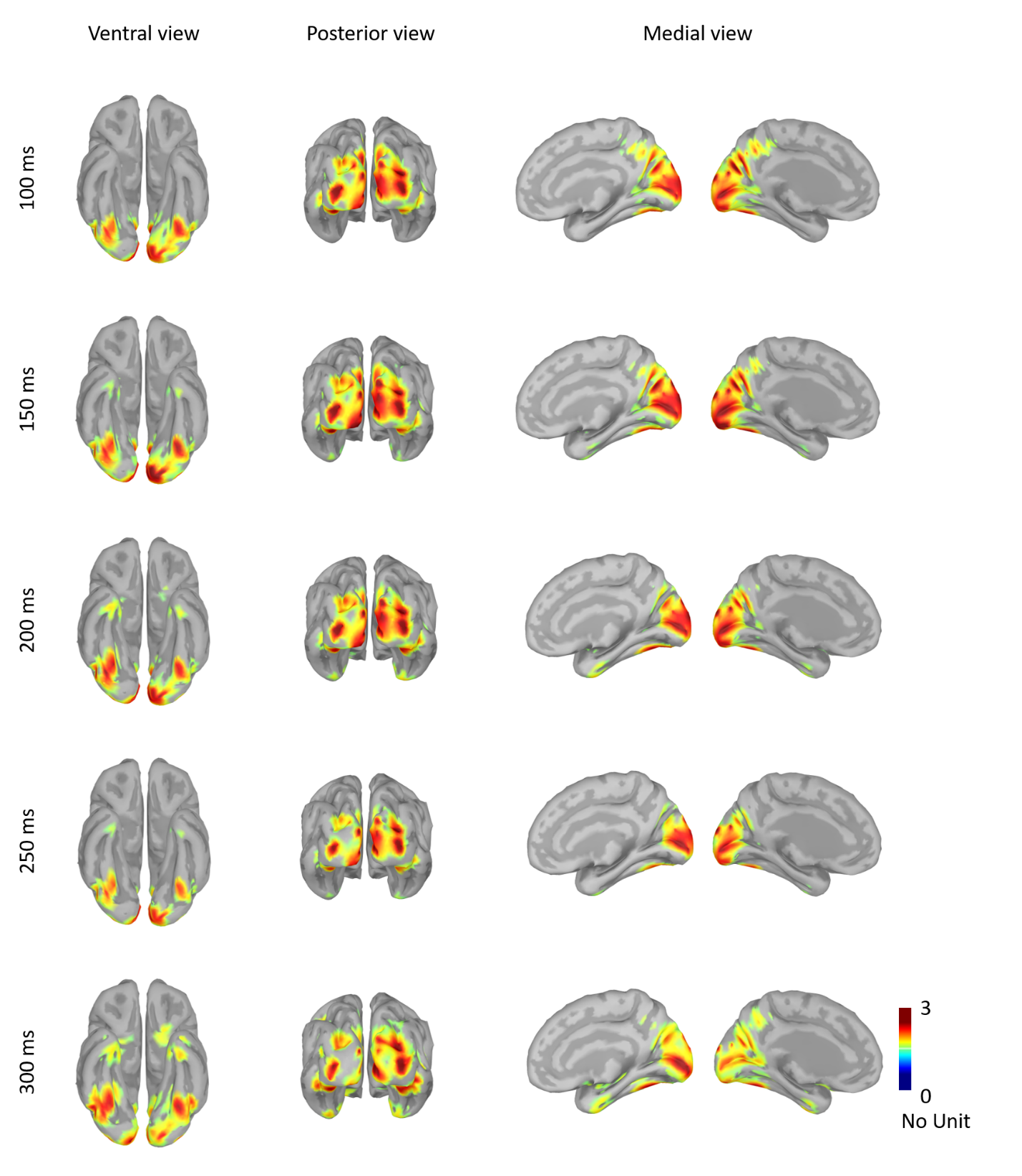


**Figure S1.** Dynamic statistical maps (dSPMs) of grand average responses to scene images from 100ms to 300ms. Activity is overlaid on the default anatomy in three views (ventral, posterior and medial).

***MEG Evoked Responses***


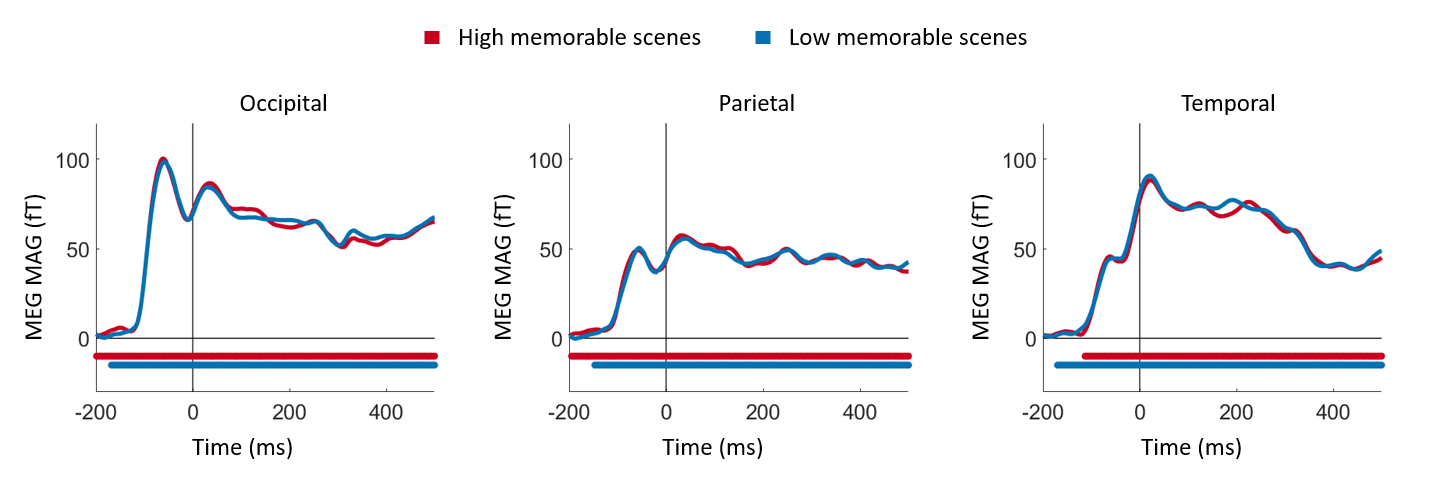


**Figure S2.** MEG evoked response fields averaged over magnetometers and subjects (N=15) in three regions, occipital, parietal, and temporal, for high memorable images (red curve), and low memorable images (blue curve). Time is from target image onset. Activity before time zero is due to the onset of the RSVP sequence. The color coded red and blue lines at the bottom of curves show the significant time points where the signal is above chance level of 0 fT. All significant statistical tests are with permutation tests using cluster defining threshold *P*<0.05, and corrected significance level *P*<0.05 (n=15).

**References**

1. Bainbridge, W.A., and Oliva, A. (2015). A toolbox and sample object perception data for equalization of natural images. Data in Brief, 5, 846-851.
2. Torralba, A., Oliva, A. (2003). Statistics of natural image categories. Netw. Comput. Neural Syst., 14, 391–412, doi:10.1088/0954-898X_14_3_302.
